# The Importance of Scientific Publications in Times of Pandemic Crisis

**DOI:** 10.6061/clinics/2020/e1895

**Published:** 2020-04-06

**Authors:** Luiz Felipe Pinho Moreira

**Affiliations:** Departamento de Cardiopneumologia, Faculdade de Medicina FMUSP, Universidade de Sao Paulo, Sao Paulo, SP, BR. Editor in Chief, CLINICS

Undoubtedly, we are living in a period of great challenge for the field of medical science. The COVID-19 epidemic is an emerging and rapidly evolving situation that has brought up scientific concepts that were no longer relevant. For a long period of time, we did not experience situations that had such a high mortality rate affecting entire populations, or diseases that followed their natural courses with little prospect of treatment or reversal of their clinical presentation.

Though we are accustomed to the concept of scientific evidence that is preferably based on large clinical trials that take years to complete, we must now rely on case studies, single cohort studies, and even the opinions of experts. However, we know that we cannot lose sight of the criteria for evidence-based medicine, and must remember to evaluate all potential biases existing in this type of scientific information without limiting its dissemination. Each piece of information always has some value, by proposing new hypotheses, expanding the debate, and evolving into new quality scientific research.

With regard to the current situation, nearly 2,000 papers with reference to COVID-19 have been registered in the PubMed database since the beginning of 2020. They include laboratory studies, clinical reports, guidelines, editorials with experts' opinions, and several observational studies. These studies show initial observational evidence regarding possible pharmacological treatments or the use of prolonged respiratory support methods as means of avoiding mortality, morbidity, or even to decrease some secondary outcomes, such as the viral load. Several potential COVID-19 treatments have been described, including drugs already in use for HIV and malaria, experimental compounds that work against an array of viruses in animal experiments, and antibody-rich plasma from people who have recovered from the disease. Other studies are dedicated to identifying groups at risk and the various factors involved—even moving toward the proposition of scores that determine the best allocation of resources and clinical interventions according to the actual status of the patients. From these data, we can also observe the rapid response of the international scientific community through the proposal and initiation of randomized multicenter clinical studies that will quickly determine the real prospect of the use of several types of treatment. Besides all of these efforts, the development of anti-viral vaccines is connecting several groups around the world, representing a necessary development for the prevention and ultimate eradication of COVID-19.

This new situation requires scientific journals to ensure the rapid publication of existing information, but at the same time to ensure its quality and identify the potential biases and limitations of the published data. As a task force of great relevance, scientific editors must filter through the existing material, while not failing to rely on the peer review process, and must immediately deliver a response to the authors and provide resources for the publication of data. In this regard, the benefits of open access publications are obvious, especially for research in which urgency and speed are so important. Unfortunately, the proportion of new scientific research being published in open access journals is disappointingly small. However, considering the urgency presented by the COVID-19 epidemic, there are some interesting initiatives to increase the openness of research, data, and publications. Nevertheless, we still see that only 30% of the articles related to this disease provide full text accessibility in the PubMed database.

The CLINICS journal, as an official publication of the Hospital das Clínicas da Faculdade de Medicina da Universidade de São Paulo (HC-FMUSP), now has the mission of expanding the scientific literature on all aspects of COVID-19. As a general and open access medical journal with an editorial board and reviewers of high scientific qualifications, that is committed to the importance and necessity of research on the current situation, CLINICS is in a prominent position of great responsibility.

